# Impact of Preoperative Diagnosis on Clinical Outcomes of Oblique Lateral Interbody Fusion for Lumbar Degenerative Disease in a Single‐institution Prospective Cohort

**DOI:** 10.1111/os.12419

**Published:** 2019-02-14

**Authors:** Sam Yeol Chang, Yunjin Nam, Jeongik Lee, Bong‐Soon Chang, Choo‐Ki Lee, Hyoungmin Kim

**Affiliations:** ^1^ Department of Orthopaedic Surgery Seoul National University Hospital Seoul Republic of Korea

**Keywords:** Oblique lateral interbody fusion, Spinal stenosis, Spondylolisthesis, Subsidence, Substantial clinical benefit

## Abstract

**Objectives:**

Oblique lateral interbody fusion is considered a useful surgical option for various lumbar degenerative diseases with favorable clinical results and few complications. However, clinical outcomes following oblique lateral interbody fusion stratified according to the preoperative diagnosis have not been fully evaluated in a large cohort. The purpose of the present study was to evaluate the clinical outcomes following oblique lateral interbody fusion for lumbar degenerative disease and to identify differences in outcomes when stratified according to preoperative diagnosis.

**Methods:**

All patients receiving oblique lateral interbody fusion for lumbar degenerative diseases were included in the current study and were stratified into four diagnostic groups: (i) degenerative spondylolisthesis; (ii) spondylolytic spondylolisthesis; (iii) spinal stenosis without spondylolisthesis and instability; and (iv) deformity. Clinical outcomes were assessed using multiple patient‐reported questionnaires. Radiologic outcomes, including cage subsidence and completion of fusion, were also evaluated.

**Results:**

Overall, 169 patients with 262 operative levels were included in the study. All clinical scoring items showed significant improvement at 1 year postoperatively for all diagnostic groups. Net and percent improvement, and a proportion of patients reaching a threshold for substantial clinical benefit were not significantly different between the diagnostic groups in all scoring items, except for lower extremity radiating pain of the deformity group. Although the deformity group had the highest overall complication rate, neurologic complications were more frequent in the spondylolytic spondylolisthesis group. The rate of complete fusion and cage subsidence for individual levels at 1 year postoperatively was 62.7% and 32.6% respectively, with no significant difference between the diagnostic groups.

**Conclusions:**

The large single‐institution prospective cohort of the present study showed favorable clinical outcomes following oblique lateral interbody fusion for lumbar degenerative disease, even in spinal stenosis without spondylolisthesis and instability.

## Introduction

Oblique lateral interbody fusion (OLIF) is considered a useful surgical method for the treatment of various lumbar degenerative diseases, with favorable clinical results and few early complications^1^, ^2^, ^3^, ^4^, ^5^, ^6^. Mechanisms of symptom improvement following OLIF include indirect decompression of the spinal canal through restoration of intervertebral disc height and reduction of spondylolisthesis, stabilization of segmental spinal instability, and gradual remodeling of the spinal canal following stabilization^7^, ^8^.

Conditions such as spinal stenosis without dynamic instability or disc height loss, lateral recess stenosis, and facet joint cysts are less likely to benefit from the mechanisms mentioned above and, therefore, have been regarded as relative contraindications for indirect decompression in previous studies^9^, ^10^, ^11^. Furthermore, clinical results following OLIF stratified according to the preoperative diagnosis have not been fully evaluated in a large cohort.

Researchers have been applying OLIF for different lumbar degenerative diseases since 2012, with over 300 cases treated with OLIF in the following 5 years. At first, OLIF was applied only for obvious surgical indications such as spondylolysis or degenerative spondylolisthesis. Over time, researchers broadened the indication for OLIF and experienced comparably favorable clinical results for conditions previously regarded as relative contraindications for OLIF. Thus, the present study was conducted to evaluate the clinical outcomes following OLIF for lumbar degenerative disease, and, more importantly, to compare the outcomes when stratified by preoperative diagnosis.

## Methods

This prospective study included all consecutive patients receiving OLIF and percutaneous pedicle screw instrumentation for lumbar degenerative disease at the author's center from January 2013 to December 2016. The patients provided informed consent and the study was approved by the institutional review board. In general, a lumbar interbody fusion was considered in patients with lumbar degenerative diseases whose symptoms were refractory to conservative treatment for more than 6 months. During this period, OLIF was considered a primary surgical option in all patients requiring lumbar interbody fusion for lumbar degenerative diseases, except in the following cases: (i) where the retroperitoneal approach for OLIF cannot be applied due to anatomical reasons, such as adhesions from previous retroperitoneal surgery or the presence of vessels blocking the oblique corridor to the L_5_S1 disc level; and (ii) sequestration of intervertebral disc on preoperative imaging studies. These patients received direct decompressive surgical interventions such as posterior lumbar interbody fusion and were excluded from the current study. Otherwise, all patients requiring lumbar interbody fusion during the study period received OLIF regardless of the severity and anatomical cause of the spinal stenosis. Patients who had a history of prior surgery on the lumbar spine and those who underwent additional surgery on the lower extremities, such as total knee replacement arthroplasty, within 1 year after OLIF surgery were excluded from the study, as this may have affected the clinical scores, especially walking ability and pain in the lower extremities.

The preoperative diagnoses of the study patients were stratified into four categories: (i) degenerative spondylolisthesis with central and/or foraminal stenosis (DS group); (ii) spondylolytic spondylolisthesis with central and/or foraminal stenosis (SS group); (iii) spinal stenosis without spondylolisthesis and dynamic instability (ST group); and (iv) degenerative lumbar deformity such as degenerative scoliosis and kyphosis (DF group). Patients with no spondylolisthesis and segmental instability on simple radiographs were included in the ST group, regardless of the disc height loss and the anatomical cause of the stenosis.

Prior to surgery, patients were required to complete several patient‐reported questionnaires, including the Oswestry disability index (ODI)^12^, the Short Form‐36 Health Survey (SF‐36)^13^, the Japanese Orthopedic Association Back Pain Evaluation Questionnaire (JOABPEQ)^14^, and the visual analogue scale (VAS) for back pain and lower extremity radiating pain. Information on other patient factors, such as the body‐mass index and the T‐score of the lumbar spine from dual‐energy X‐ray absorptiometry scan, were also collected.

The OLIF procedure was performed using a minimally invasive anterior retroperitoneal approach with the patient in the lateral decubitus position. After splitting three layers of abdominal muscle and entering the retroperitoneal space, the ante‐psoas oblique corridor was used to expose the intervertebral disc space of L_2_‐L_5_ levels. The L_5_S_1_ level was approached between the major vessels through the retroperitoneal oblique corridor with the patient maintained in the lateral position, as described in a previous anatomic study.^15^ After meticulous discectomy and end‐plate preparation, a polyetheretherketone cage loaded with allogeneic demineralized bone matrix mixed with cancellous bone was inserted into the disc space. Autologous iliac bone graft and recombinant human bone morphogenic protein‐2 (rhBMP‐2) were not used in any patient. After moving the patient into the prone position, a percutaneous pedicle screw instrumentation was performed under intraoperative C‐arm fluoroscopic guidance. No surgical drain was used, and patients started ward ambulation 1 day after the procedure.

Patients were scheduled to visit the outpatient clinic at 3, 6 and 12 months postoperatively for outcome assessment. Clinical outcome was evaluated using the same assessment tools (SF‐36, ODI, JOABPEQ, and VAS for back pain and radiating pain) that were applied before the operation. With regards to the radiologic outcomes, the change in the degree of spondylolisthesis was evaluated using the preoperative and 1‐year postoperative simple radiographs for the DS and SS groups. The presence and grade of cage subsidence were also measured from the simple radiographs using the method described by Marchi *et al.* 16 at each outpatient clinic visit for all patients. In addition, the success of the interbody fusion procedure, defined as the presence of bridging trabecular bone between adjacent endplates^17^, was assessed using CT 1 year postoperatively in selected patients who agreed to take the examination. Information on the occurrence of intraoperative and postoperative complications was also collected and retrospectively reviewed.

For statistical analysis, paired *t*‐tests and repeated measures ANOVA with *post hoc* Bonferroni adjustments were applied to identify the improvement in clinical scores over time. One‐way ANOVA with a *post‐hoc* Tukey test and the χ^2^‐test were used to compare clinical outcomes stratified by preoperative diagnosis. To identify the difference in fusion and subsidence rates between the diagnostic groups, the χ^2^‐test and the Fisher exact test were applied. Student's *t*‐test was used to compare the clinical scores of groups with and without incomplete interbody fusion or cage subsidence. SPSS Statistics, version 25.0 (IBM, Armonk, NY, USA) was used for the statistical analysis, and a *P*‐value of <0.05 was considered statistically significant.

## Results

A total of 169 patients with 262 operative levels were included in the present study. Information on demographics and operative levels of the current cohort is presented in Table 1. The only significant demographic difference between the diagnostic groups was the mean age at surgery between the SS group and the ST group. The SS group had a markedly larger proportion of L_5_S_1_ level operations (43.9% of all levels operated in the SS group), compared to the other diagnostic groups. As anticipated, the DF group had more operated levels per patient (3.53 levels per patient) compared to other groups.

**Table 1:** Demographic data and operative levels stratified by preoperative diagnosis

Indexes		Total (*n* = 163)	DS (*n* = 93)	SS (*n* = 37)	ST (*n* = 22)	DF (*n* = 17)	*P*‐value
Age (mean ± SD, years)		67.7 ± 10.9	68.2 ± 9.8	59.8* ± 16.4	70.5* ± 5.9	64.4 ± 5.7	0.031†
Sex, M:F (*n*)		61:108	32:61	6:31	13:9	2:15	0.142‡
BMI (mean ± SD,kg/m^2^)		24.7 ± 3.0	24.1 ± 2.4	25.1 ± 3.6	25.0 ± 2.7	25.6 ± 4.3	0.695†
T‐score (mean ± SD)		−0.2 ± 1.8	−0.4 ± 1.7	−0.6 ± 1.7	0.1 ± 2.2	0.3 ± 1.4	0.517†
Operative levels (*n*, %)	L_1–2_	3 (1.1)	0 (0.0)	0 (0.0)	0 (0.0)	3 (5.0)	
	L_2–3_	26 (9.9)	8 (6.3)	0 (0.0)	2 (6.1)	16 (26.7)	
	L_3–4_	63 (24.0)	30 (23.4)	4 (9.8)	12 (36.4)	17 (28.3)	
	L_4–5_	131 (50.0)	79 (61.7)	19 (46.3)	16 (48.5)	17 (28.3)	
	L_5_S_1_	39 (14.9)	11 (8.6)	18 (43.9)	3 (9.1)	7 (11.7)	
	Total	262 (100)	128 (100)	41 (100)	33 (100)	60 (100)	
Level/patient (*n*)		1.55	1.38	1.11	1.50	3.53	

* SS and ST groups show significant difference in mean age in one‐way ANOVA test.

† One‐way ANOVA test; *P*‐value.

‡ Pearson χ^2^‐test; *P*‐value.

BMI, body‐mass index; DF, deformity; DS, degenerative spondylolisthesis; SD, standard deviation; SS, spondylolytic spondylolisthesis; ST, spinal stenosis.

**Figure 1:**
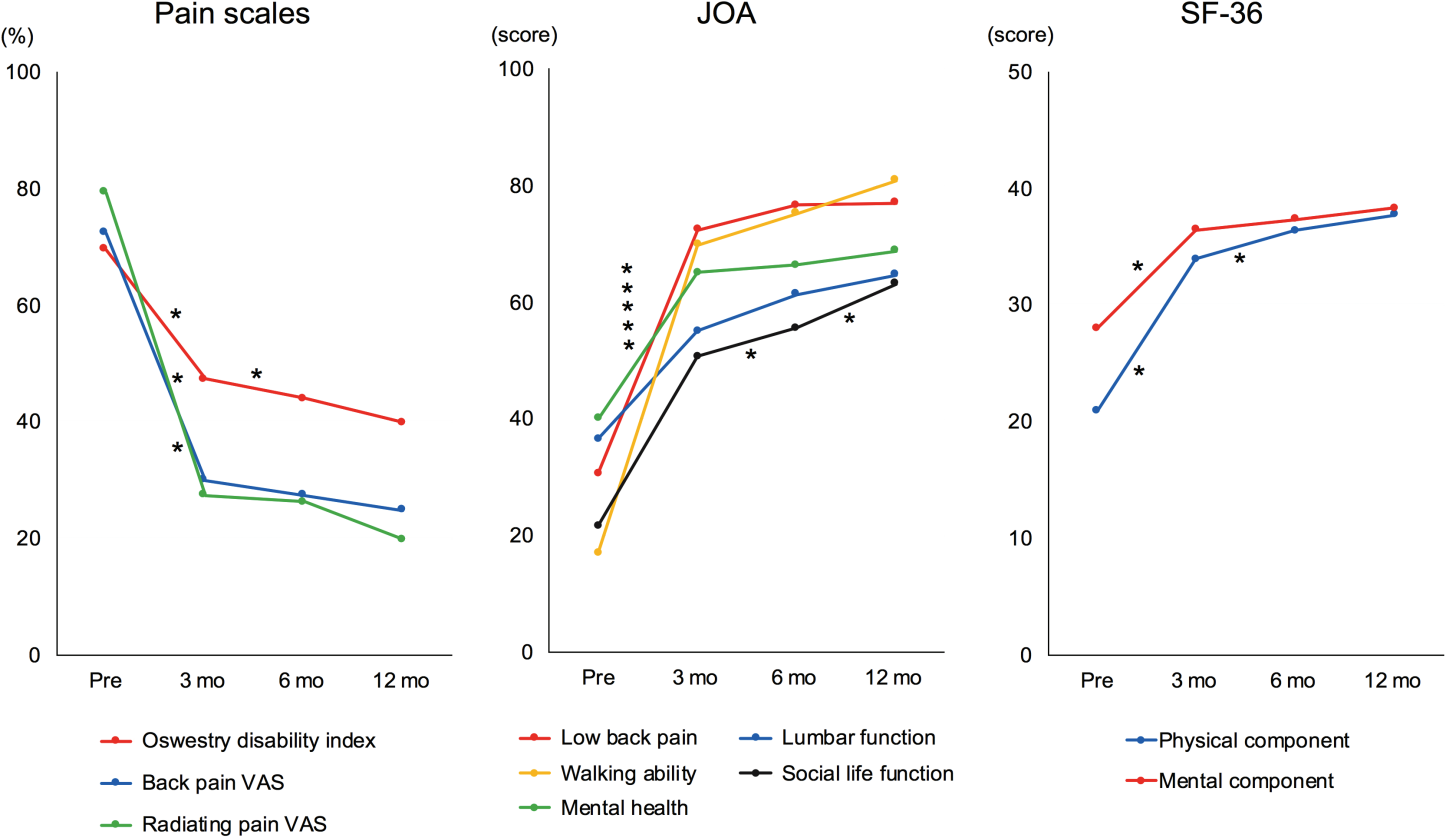
Serial change of clinical scores following oblique lateral interbody fusion (OLIF). JOA, Japanese Orthopedic Association Back Pain Questionnaire; mo, months; Pre, preoperative; SF‐36, Short Form‐36 Health Survey; VAS, visual analogue scale. *An interval with statistically significant change (by paired *t*‐test).

**Figure 2:**
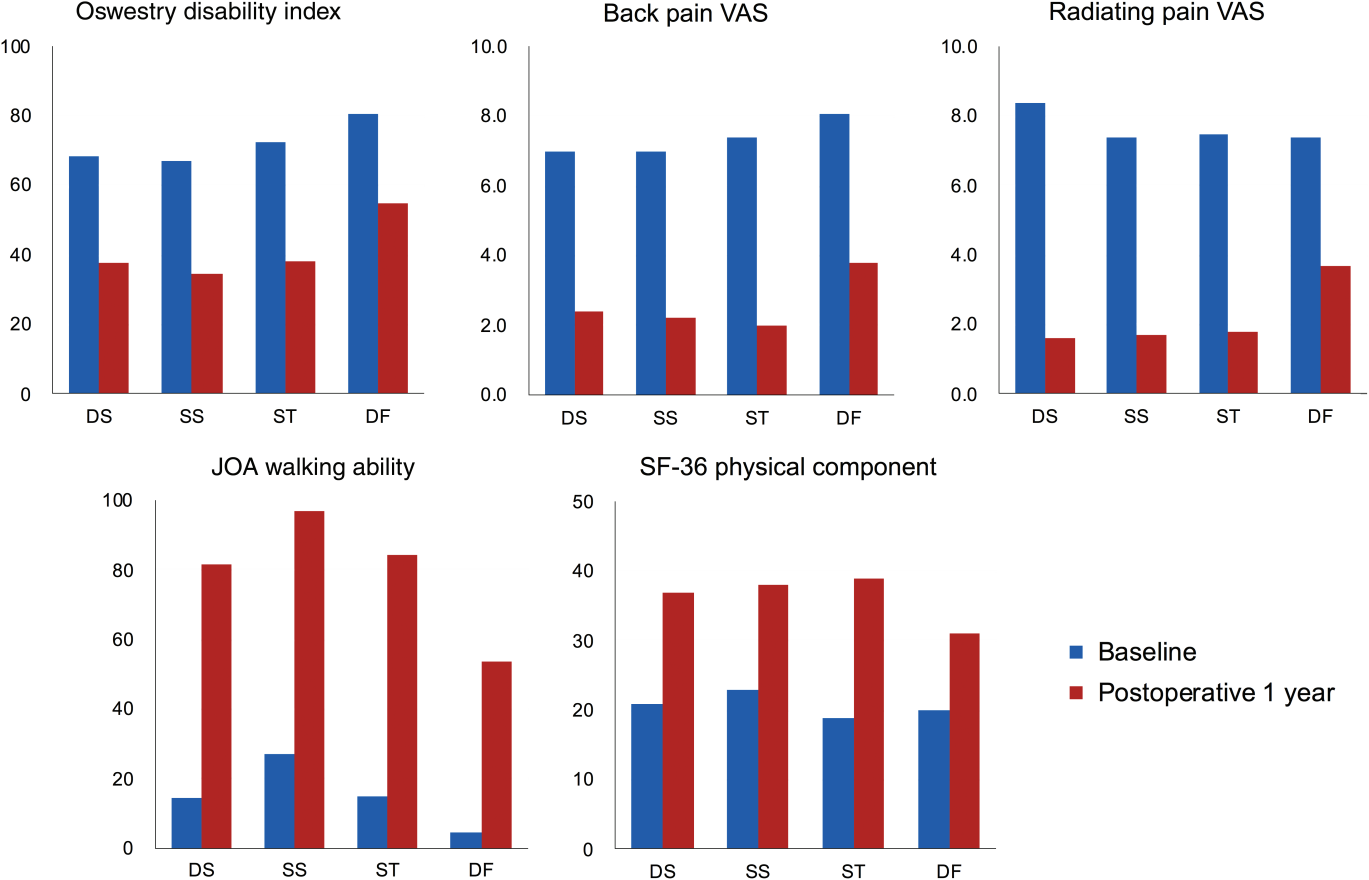
Comparison of baseline and 1‐year postoperative clinical scores after stratification into preoperative diagnosis. DF, deformity group; DS, degenerative spondylolisthesis group; SS, spondylolytic spondylolisthesis group; ST, spinal stenosis group.

All the examined clinical scores showed a trend for improvement during the 1‐year follow‐up period, which was most significant within 3 months after surgery (Fig. 1). After stratification into preoperative diagnosis, the only difference in preoperative baseline scores was walking ability assessed by the JOABPEQ between the SS and DF groups (Table 2). The baseline scores of all evaluated items showed a significant improvement 1 year postoperatively when examined by paired *t*‐test, with walking ability in JOABPEQ showing the largest improvement among all groups (Table 2 and Fig. 2). Comparison of net and percent improvement of scores 1 year postoperatively between the diagnostic groups showed that there was a statistically significant difference in improvement of radiating pain assessed by the VAS between the DF group and the DS, DF, and SS groups (Table 3). DS, SS, and ST groups showed no significant difference in the raw score, or the net and percent improvement of scores 1 year postoperatively for all scoring items.

**Table 2:** Baseline and postoperative 1‐year clinical scores stratified by preoperative diagnosis

Clinical scores		DS		SS		ST		DF	
		Baseline	PO 1yr	Baseline	PO 1yr	Baseline	PO 1yr	Baseline	PO 1yr
ODI		68.3 ± 14.7	37.7 ± 13.5	67.0 ± 20.8	34.4 ± 5.1	72.5 ± 17.1	38.2 ± 15.2	80.7 ± 14.8	55.0 ± 14.9
SF36 PCS		21.0 ± 12.0	38.6 ± 7.6	22.4 ± 13.1	39.0 ± 5.8	19.1 ± 11.1	39.6 ± 7.4	20.0 ± 9.8	30.9 ± 6.6
JOA	LBP	41.1 ± 32.2	74.2 ± 24.8	28.6 ± 20.6	88.6 ± 6.0	20.9 ± 15.0	75.0 ± 27.3	19.6 ± 15.2	73.2 ± 28.0
BPEQ	LF	37.5 ± 31.7	67.1 ± 23.8	48.3 ± 36.6	75.8 ± 18.2	35.3 ± 34.7	61.8 ± 30.9	17.7 ± 19.1	50.0 ± 24.8
	WA	14.6 ± 16.9	81.8 ± 28.6	27.1‡ ± 19.3	97.1 ± 3.7	14.8 ± 17.8	84.5 ± 24.5	4.5‡ ± 8.5	53.6 ± 31.0
	SLF	18.2 ± 19.0	65.0 ± 23.1	31.8 ± 24.8	71.9 ± 13.8	22.7 ± 17.8	65.1 ± 21.3	12.5 ± 10.1	45.3 ± 15.9
	MH	41.7 ± 19.0	71.5 ± 17.9	46.3 ± 27.5	73.4 ± 11.9	33.5 ± 18.5	72.9 ± 14.9	32.2 ± 20.3	50.8 ± 11.2
VAS	LBP	7.0 ± 2.2	2.4 ± 1.9	7.0 ± 3.0	2.2 ± 1.6	7.4 ± 1.4	2.0 ± 1.6	8.1 ± 1.1	3.8 ± 1.6
	Rp	8.4 ± 1.7	1.6 ± 1.6	7.4 ± 1.6	1.7 ± 1.4	7.5 ± 2.0	1.8 ± 1.7	7.4 ± 1.6	3.7 ± 2.0

All scores in the table are shown as mean ± standard deviation.

All baseline scores showed significant improvement at 1 year postoperatively for paired *t*‐tests (*P* < 0.05).

‡ SS and DF groups show significant difference in baseline walking ability for a one‐way ANOVA test with a *post‐hoc* Tukey test (*P* = 0.031).

DF, deformity; DS, degenerative spondylolisthesis; JOABPEQ, Japan Orthopedic Association Back Pain Evaluation Questionnaire; LBP, low back pain; LF, lumbar function; MH, mental health; ODI, Oswestry disability index; PO 1yr, postoperative 1 year; Rp, radiating pain; SF36 PCS, short‐form 36 health survey physical component score; SLF, social life function; SS, spondylolytic spondylolisthesis; ST, spinal stenosis; VAS, visual analogue scale; WA, walking ability.

**Table 3:** Net and percent improvement of clinical scores 1‐year postoperatively, stratified by preoperative diagnosis

Clinical scores		Net improvement (points, mean ± SD)				Percent improvement (%, mean ± SD)			
		DS	SS	ST	DF	DS	SS	ST	DF
ODI		30.1 ± 14.5	32.6 ± 22.6	35.2 ± 16.3	25.8 ± 18.6	44.0 ± 19.5	44.8 ± 16.7	48.2 ± 14.9	30.9 ± 18.9
SF36 PCS		18.0 ± 11.2	16.6 ± 11.4	20.8 ± 6.4	10.9 ± 8.2	44.7 ± 27.1	40.1 ± 28.5	53.8 ± 16.0	34.5 ± 30.2
JOA	LBP	32.9 ± 41.0	60.0 ± 25.0	53.6 ± 54.5	53.6 ± 31.2	25.0 ± 79.8	68.8 ± 28.8	74.5 ± 42.1	65.5 ± 32.3
BPEQ	LF	28.4 ± 32.8	27.5 ± 29.9	30.6 ± 30.2	32.3 ± 24.1	42.5 ± 49.5	40.2 ± 42.6	47.1 ± 51.9	66.4 ± 32.7
	WA	68.6 ± 35.6	70.0 ± 21.0	70.2 ± 25.6	49.1 ± 31.0	72.4 ± 67.9	71.8 ± 20.1	83.4 ± 42.1	84.6 ± 35.1
	SLF	47.6 ± 23.5	40.0 ± 24.1	42.8 ± 21.7	32.7 ± 10.4	72.8 ± 23.4	55.8 ± 33.2	65.0 ± 31.1	75.0 ± 15.9
	MH	32.8 ± 26.3	27.1 ± 30.1	38.6 ± 17.1	18.7 ± 19.1	38.2 ± 50.7	35.0 ± 38.6	43.8 ± 34.9	37.7 ± 43.1
VAS	LBP	4.6 ± 2.7	4.8 ± 3.5	5.4 ± 2.3	4.4 ± 1.5	62.8 ± 28.7	72.3 ± 22.7	71.5 ± 23.0	54.1 ± 19.1
	Rp	6.7* ± 1.8	5.7* ± 2.1	5.6 ± 1.7	3.4* ± 1.3	81.8* ± 17.2	78.2* ± 20.5	76.4 ± 20.6	47.6* ± 22.5

* DF group showing less improvement in radiating pain VAS, compared to DS and SS groups. (*P* < 0.05 in one‐way ANOVA with post‐hoc Tukey test.)

LBP, low back pain; LBP, low back pain; LF, lumbar function; MH, mental health; ODI, Oswestry disability index; PO 1yr, postoperative 1 year; Rp, radiating pain; SD, standard deviation; SF36 PCS, short‐form 36 health survey physical component score; SLF, social life function; SS, spondylolytic spondylolisthesis; ST, spinal stenosis; VAS, visual analogue scale; WA, walking ability.

**Figure 3:**
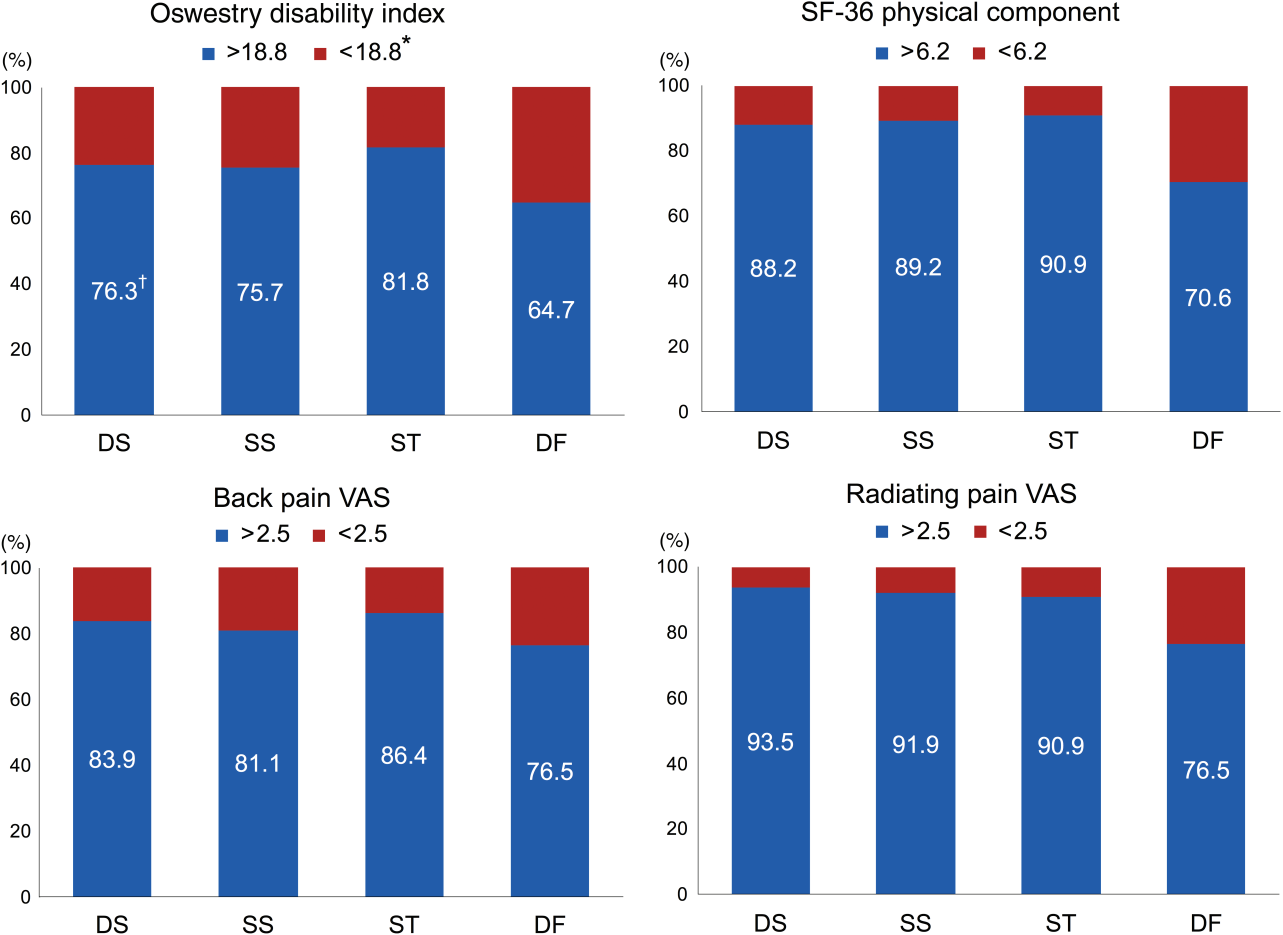
Proportion of patients reaching a threshold for substantial clinical benefit, after stratification into preoperative diagnosis. *Reference values based on net improvement of scores in each questionnaire. ^†^Percentages of patients achieving substantial clinical benefit.

The proportion of patients reaching substantial clinical benefit (SCB), based on the net improvement of the ODI, physical component score of SF‐36, and VAS for lower back and radiating pain 1 year postoperatively, are described in Fig. 3. Reference values for SCB were adapted from the study by Glassman *et al.* 18 which defined SCB following lumbar arthrodesis. The proportion of patients achieving SCB for radiating pain assessed by the VAS was significantly smaller for the DF group compared to the other groups. The other three groups (DS, SS, and ST) showed no significant difference in the proportion of patients attaining SCB for all examined items.

Perioperative complications occurred in 27 of 169 (16.6%) cases (Table 4). Neurologic symptoms such as aggravation or insufficient relief of lower extremity radiating pain or weakness after the operation, which required additional treatment within postoperative 2 weeks, were observed in 14 of 169 (8.6%) cases (Table 5). Among these patients, 11 of 14 showed symptom improvement after epidural steroid injection and did not require additional surgery, leaving only 3 patients (1.8%) who failed to achieve successful symptom relief with indirect decompression. The reasons for the failure of indirect decompression in these cases were: (i) a malposition of the interbody cage resulting in the encroachment of the contralateral neural foramen; (ii) a missed diagnosis of a sequestered disc; and (iii) excessive postoperative segmental lordosis causing the narrowing of the neural foramen at the L_5_S_1_ level. Although the proportion of patients with overall perioperative complications was highest in the DF group (4 of 17 patients, 23.5%), neurologic complications occurred more frequently in the SS group (5 of 37 patients, 13.5%).

**Table 4:** Overall perioperative complications

Timing	Complications	Number (%)	
Intraoperative	Vessel injury	3 (1.8)	2: iliac vessel, 1: segmental artery
Early PO	Epidural block (<2 weeks)	11 (6.7)	
	Posterior decompression	3 (1.8)	
	Infection	1 (0.6)	Revision posterior lumbar interbody fusion done
	Psoas weakness	1 (0.6)	
Late PO	Adjacent segmental disease	6 (3.6)	3: at PO 1 year, 2: at 2 years, 1: at 5 years
	Screw loosening	2 (1.2)	1: subclinical, 1: revision at PO 10 months
Total		27 (16.6)	

Proportion of patients with complications among total 169 patients.

PO, postoperative.

**Table 5:** Number of patients with overall and neurologic perioperative complications according to preoperative diagnosis

Diagnostic groups	Overall complications (*n*, %)	Neurologic complications (*n*, %)
DS (*n* = 93)	15 (16.1*)	4 (4.3*)
SS (*n* = 37)	6 (16.2*)	5 (13.5*)
ST (*n* = 22)	2 (9.1*)	1 (4.5*)
DF (*n* = 17)	4 (23.5*)	2 (11.8*)
Total (*n* = 169)	27 (16.6†)	14 (8.6†)

* Proportion of patients with complications among patients in the subgroup.

† Proportion of patients with complications among total 169 patients.

DF, deformity; DS, degenerative spondylolisthesis; SS, spondylolytic spondylolisthesis; ST, stenosis without instability.

As a radiological parameter, the degree of spondylolisthesis was evaluated using the preoperative and postoperative 1‐year simple radiographs for DS and SS groups. All 93 patients in the DS group showed grade I spondylolisthesis (0%–25% slippage) preoperatively. Among these 93 patients, 76 (81.7%) patients showed a complete reduction of spondylolisthesis, while the other 17 (18.3%) patients had grade I spondylolisthesis remaining 1 year postoperatively. For 37 patients in the SS group, 28 patients had grade I spondylolisthesis, and the other 9 had grade II spondylolisthesis (25%–50% slippage) preoperatively. There were no patients with grade III to V spondylolisthesis (>50% slippage). Among these patients, 27 (73.0%) patients had improvement of one degree of spondylolisthesis, while the other 10 (27.0%) were classified into the same degree (I or II) of spondylolisthesis as preoperative status 1 year postoperatively.

Completion of interbody fusion, defined as the presence of bridging trabecular bone, was evaluated for 94 of 169 patients who had a CT examination 1 year postoperatively. Among 94 patients examined, 51 (54.3%) had all operated levels completely fused, while the other 43 (46.7%) had at least 1 level with incomplete fusion. For individual levels, complete interbody fusion was observed in 84 of 134 (62.7%) examined levels. No statistically significant difference in fusion completion rate was observed between the diagnostic groups (Fig. 5). Furthermore, for the whole cohort, a group of patients with at least 1 level of incomplete fusion did not show any statistical difference for all clinical scores when compared to a group of patients who had complete interbody fusion in all levels.

Subsidence of the interbody cage 1 year postoperatively was evaluated in all patients except for 1 patient who received revision posterior lumbar interbody fusion due to postoperative infection following single‐level OLIF at the L_5_S_1_ level. Cage subsidence was observed in 85 of 261 (32.6%) operated levels, and 62 of 168 (36.9%) patients had at least 1 level with cage subsidence of grade 1 or more. When stratified by preoperative diagnosis and operative levels, the SS group and L_5_S_1_ level had the smallest proportion of levels with cage subsidence (24.4% and 13.2%, respectively) (Fig. 6). For the whole cohort, a group of patients with at least 1 level of cage subsidence had poor clinical scores 1 year postoperatively compared to a group of patients with no subsidence when examined by Student *t*‐test: higher ODI (45.4 to 33.8, *P* = 0.02) and lower SF‐36 physical component score (33.6 to 41.4, *P* = 0.01).

## Discussion

Despite the favorable clinical outcomes following indirect decompression in various lumbar degenerative diseases, the effectiveness of indirect decompression has not been clarified for specific conditions. In particular, there is little evidence regarding the clinical outcomes of OLIF in spinal stenosis without spondylolisthesis and dynamic segmental instability. The large prospective cohort of the present study included 22 cases of spinal stenosis without spondylolisthesis and instability, designated as the ST group. This group consisted of various degrees of stenosis, including 8 patients with severe (grade C) and 7 with extreme (grade D) stenosis, according to the qualitative grading system introduced by Schizas *et al*.^19^. Regarding the final raw score, and net and percent improvement of clinical scores, the ST group showed no significant differences when compared to the DS and SS groups, which are considered typical indications for OLIF. The proportion of ST group patients reaching the threshold for SCB was also not statistically inferior to the other groups. These results suggest that OLIF can be a useful surgical method even in cases of spinal stenosis without spondylolisthesis and instability.

The abovementioned patients can be further divided into two groups: those with and without loss of disc height. Among 22 patients in the ST group, 14 showed no loss of disc height compared to adjacent levels in preoperative radiologic studies. However, the mean disc height in these 14 patients was further increased after OLIF (anterior height from 12.3 to 14.4 mm, posterior disc height from 7.9 to 8.5 mm), which was statistically significant when examined by paired *t‐*test (*P* = 0.003). These findings suggest that spinal stenosis patients without instability and disc height loss, for whom indirect decompression is expected to be less effective, may also benefit from the stabilization and further elevation of disc height.

Because fusion surgery is known to increase the risk of adjacent level degeneration, some surgeons might object to the application of OLIF and prefer posterior decompression surgery without fusion for stenosis patients without instability and disc height loss. However, because posterior direct decompression has several disadvantages, such as epidural bleeding and scarring, a simple conclusion on the more appropriate surgical choice cannot be drawn at this time. Well‐designed prospective randomized control studies with long‐term follow up comparing different clinical outcomes of posterior decompression without fusion and OLIF for these conditions are required for more clarity.

In the current study, 14 of 169 patients complained of neurologic symptoms such as aggravation or insufficient relief of lower extremity radiating pain or weakness after the operation, which required additional treatment. The occurrence rate of such complications was highest in the SS group (Table 5). This result is believed to be associated with the higher proportion of L_5_S_1_ level in the SS group (43.9%), of which a poorer clinical outcome was also observed after indirect decompression in previous studies^1^, ^8^, ^11^. Among these 14 patients with neurologic symptoms, only 3 patients received additional posterior direct decompression surgery for reasons described in the result section. In such patients with a huge extruded or sequestered disc, large posterior osteophytes from the superior articular process that may aggravate foraminal narrowing, direct decompression is an optimal surgical method. It is also noteworthy that the severity of the preoperative stenosis was not a deciding factor for the addition of direct decompression surgery in this study cohort. However, it is beyond the scope of this study to evaluate the effectiveness of indirect decompression in severe spinal stenosis.

**Figure 4:**
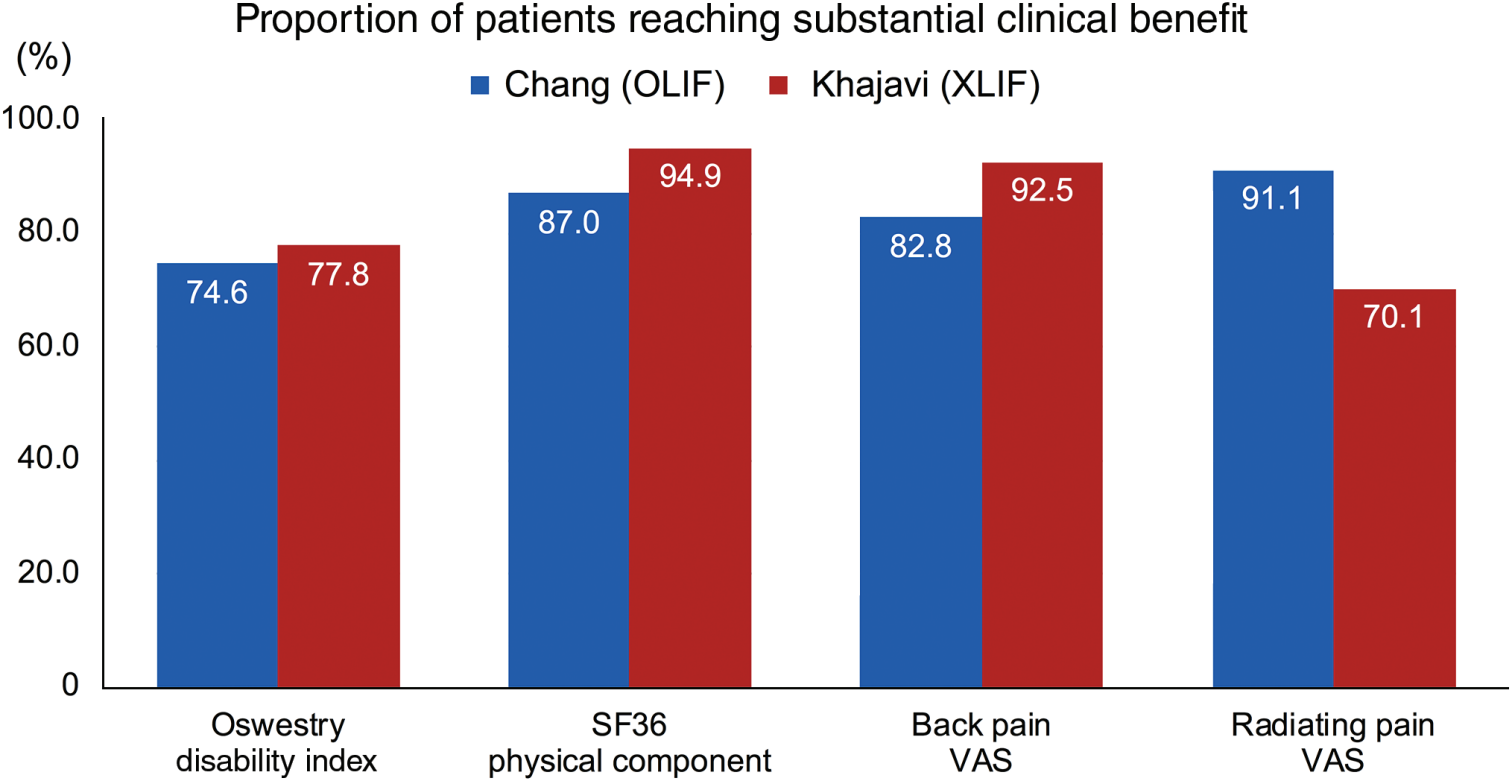
Proportion of patients reaching substantial clinical benefit following lumbar interbody fusion for degenerative spondylolisthesis: Comparison between the oblique lateral interbody fusion and extreme lateral interbody fusion. OLIF, oblique lateral interbody fusion; XLIF, extreme lateral interbody fusion.

The whole cohort of the present study showed favorable clinical outcomes compared to other clinical studies applying the concept of SCB. In a study by Khajavi *et al*., 70.7%–84.0% of patients who received extreme lateral interbody fusion (XLIF) for lumbar degenerative disease achieved the threshold for SCB in the ODI, a numerical rating scale for back and radiating pain^20^. These numbers cannot be directly compared becaue the inclusion criteria and the patient composition of the two studies are different. For patients with degenerative spondylolisthesis, which is a common group of the two studies, the proportion of patients achieving the threshold for SCB is compared in Fig. 4.

**Figure 5:**
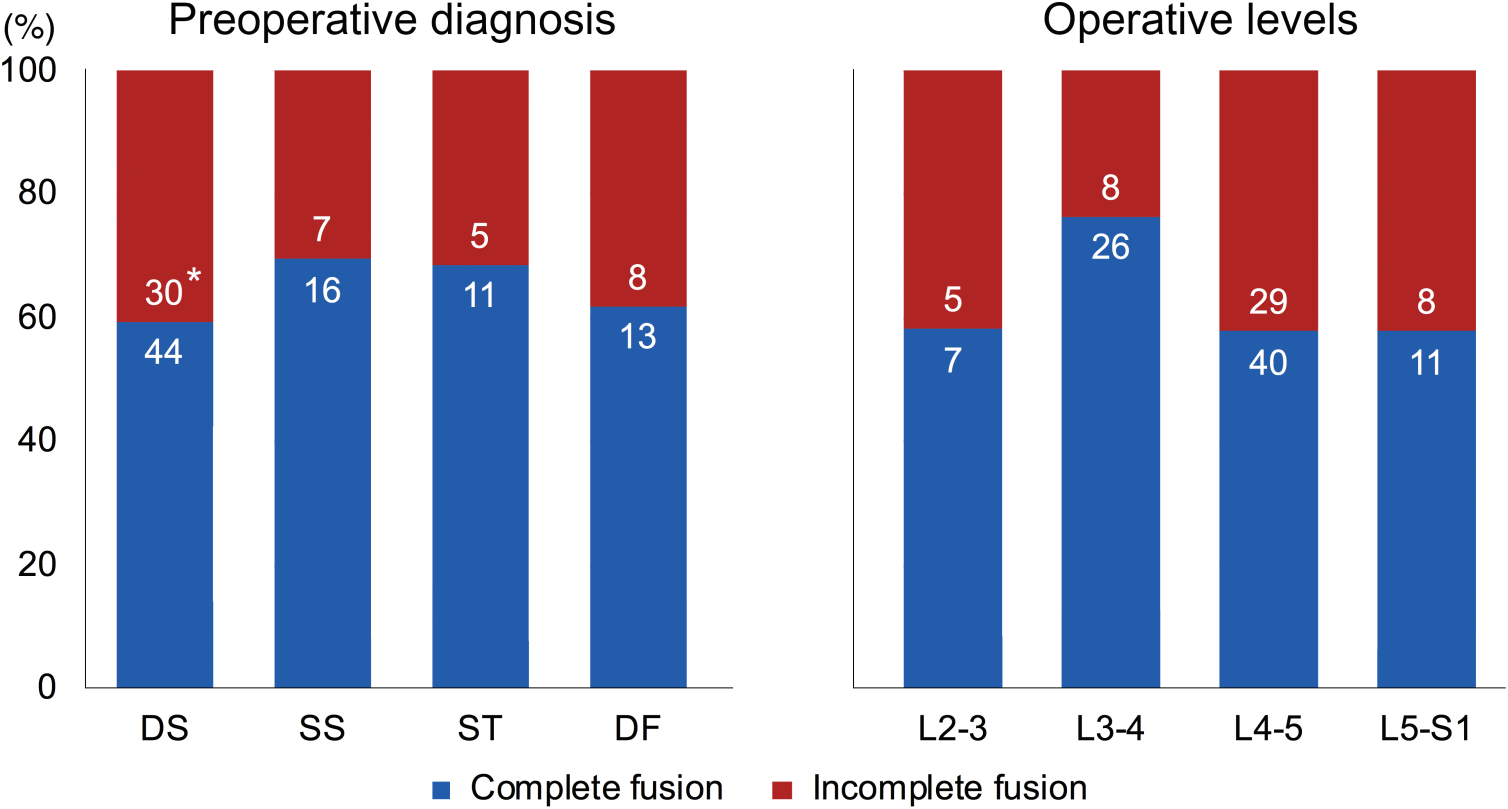
Fusion status of individual levels 1 year postoperatively, stratified by preoperative diagnosis and operative levels. *Numbers of levels with complete or incomplete interbody fusion.

**Figure 6:**
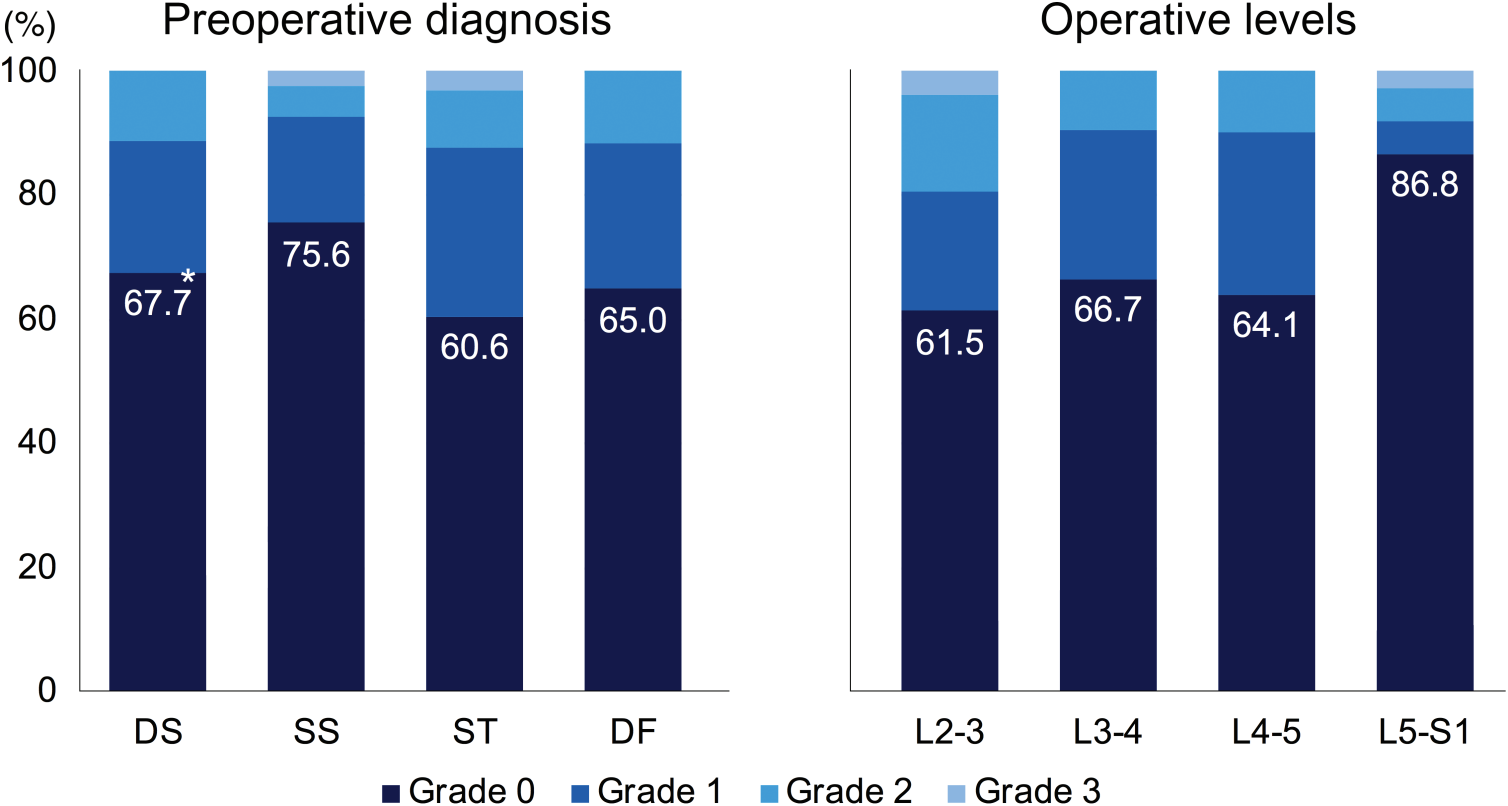
Distribution of the grading for cage subsidence 1 year postoperatively, stratified by preoperative diagnosis and operative levels. *Percentage of levels without cage subsidence.

One of the strengths of the present study lies in the observation of a trend in improvement over time. The present prospective patient cohort showed a consistent trend of improvement in clinical scores during the follow‐up period up to 1 year postoperatively. Although the trend for improvement was observed throughout the entire 12‐month period, the most statistically significant improvement occurred within 3 months after surgery for all scoring items. Clinical improvement in the early postoperative period seems to be from the immediate effect of stabilization with disc height restoration, while improvement in the later stage appears to be from the impact of gradual remodeling of the spinal canal, which is often observed in the follow‐up MRI studies.

Completion of interbody fusion was identified in 84 of 134 (62.7%) levels evaluated in the CT study 1 year postoperatively. This result can be compared to the study by Malham *et al*.^10^, which reports a solid fusion occurring in 85 of 122 (69.7%) patients 12 months postoperatively following XLIF. Other studies^3^, ^5^, ^21^, ^22^ have reported relatively higher fusion rates after indirect decompression (XLIF, OLIF) than the present study, which may result from differences in the composition of bone substitute materials inserted into the interbody cages. Some of these studies with higher fusion rates routinely used rhBMP‐2 for interbody fusion, which was not used for the patients in the present study.

Cage subsidence was observed in 32.6% of operative levels, which is similar to or slightly higher than that of previous studies^4^. The relatively higher subsidence rate in the current study may be related to the technical effort required to insert cages having the greatest height possible for disc height restoration in earlier stages of our institution's experience with OLIF procedures. The authors later modified this technical approach to inserting cages with a height just sufficient to provide disc height elevation and segmental stability, based on the observations that stabilization is the primary mechanism of symptom improvement in the early stage after the OLIF procedure and cage subsidence is associated with poor clinical outcomes.

There are several limitations to the present study. First, although the size of the entire cohort is considerably large, the sample sizes of the individual diagnostic groups, especially the ST and DF groups, were small, therefore limiting statistical power. Second, because only clinical outcomes within 12 months after surgery were evaluated in the present study, the long‐term clinical results of OLIF procedures are still questionable. Finally, the clinical outcomes of OLIF achieved in this study cannot be directly compared with other surgical methods applicable to lumbar degenerative diseases. Despite these limitations, the findings of the current study suggest the possibility of expansion of surgical indications for OLIF in the lumbar degenerative diseases.

In conclusion, the large prospective single‐institution cohort in the current study showed favorable clinical outcomes following OLIF for the lumbar degenerative disease. OLIF may represent an effective surgical option even in spinal stenosis without spondylolisthesis and segmental instability.
